# JAK2 inhibitor CEP-33779 prevents mouse oocyte maturation *in vitro*

**DOI:** 10.1042/BSR20170642

**Published:** 2017-07-12

**Authors:** Changli Wu, Rong Li, Haibing Luo, Mingfeng Xu, Xiujuan Zhang

**Affiliations:** 1 Department of Physiology, Guangdong Medical University, Zhanjiang, Guangdong, People’s Republic of China; 2 Department of Pathophysiology, Guangdong Medical University, Zhanjiang, Guangdong, People’s Republic of China

**Keywords:** CEP-33779, JAK2, microfilament, oocyte maturation

## Abstract

The inhibitor CEP-33779 is a specific selective inhibitor of Janus kinase 2 (JAK2). In most somatic cells, JAK2 plays essential roles in cellular signal transduction and in the regulation of cell cycle. Little is known regarding the effects of JAK2 on mammalian oocyte maturation. In the present study, we investigated the effects of CEP-33779 on mouse oocytes’ meiosis and the possible mechanisms of JAK2 during mouse oocyte maturation. We detected the distribution of JAK2 during the mouse oocyte maturation. The results showed that JAK2 was mainly distributed in the cytoplasm during maturation. We cultured mouse oocytes with CEP-33779, examined the maturation rate, spindle morphology, and organization of microfilaments during the mouse oocyte maturation. While the rate of germinal vesicle breakdown (GVBD) did not differ between the treated and control groups, the rate of oocyte maturation decreased significantly when treated with CEP-33779. The rate of maturation was 21.14% in treated group and was 81.44% in control group. The results show that CEP-33779 inhibits the maturation of mouse oocytes. There was no obvious difference in the meiotic spindle morphology between the treated and control groups. The results show that CEP-33779 treatment did not disrupt the reorganization of microtubules. The microfilament observation shows that the microfilament did not form actin cap and the spindle stayed at the center of the oocyte in the treated group. CEP-33779 treatment inhibited the maturation of mouse oocytes which might be because of the disruption of formation of the actin cap. These results suggest that JAK2 regulated the microfilaments aggregation during the mouse oocyte maturation.

## Introduction

Mammalian oocyte maturation involves an ordered series of cellular events, including germinal vesicle breakdown (GVBD), chromosome condensation, spindle formation, and migration. The asymmetric separation of the cytoplasm and accurate symmetric segregation of the maternal genome produce polar bodies and a competent, fertilizable oocyte. These asymmetric divisions are regulated by microtubules and actin filaments [1,2]. The meiotic spindle migrates from central areas to the periphery of an oocyte in an actin-dependent manner. The actin aggregated to form an actin cap which marks the site of polar body extrusion [3,4].

Janus kinase 2 (JAK2) belongs to the non-receptor tyrosine kinase family [5]. JAK2 is widely distributed in the cytoplasm of a variety of somatic cells, involved in cell-cycle regulation, apoptosis [6], mitotic chromosome recombination [7], genetic instability, and heterochromatin changes [8], and other biological processes. Selective knockdown of JAK2 in mice can result in embryonic anemia and death at approximately 12.5 days [9]. In most of the somatic cells, JAK2 mainly exists in the cytoplasm and its function is dependent on its substrate STAT. But in a small number of specific cells, JAK2 is present in the nucleus. JAK2 exists in the nucleus of hematopoietic cells, which can activate the transcription of H3Y41 through phosphorylation of the histone protein [10]. The shuttle between the cytoplasm and the nucleus of JAK2 is mainly regulated by ubiquitination of JAK2 [11]. In mouse mature oocyte and early cleavage stage embryo, JAK2 localizes to the chromosomes [12]. However, the localization and functions of JAK2 on the asymmetric division of mouse oocytes are still unclear. The present study was aimed to investigate the role of JAK2 in the meiosis of mouse oocytes.

CEP-33779 is a highly selective, small-molecule inhibitor of JAK2 [13,14]. CEP-33779, as a specific inhibitor of JAK2, has been used in preclinical studies of some diseases. CEP-33779 depleted the autoreactive plasma cells and treated lupus nephritis in mice [15]. CEP-33779 ablated disease in two different mouse models of rheumatoid arthritis [13]. Administration of CEP-33779 resulted in significant inhibition of tumor growth in an AOM/DSS-induced model of colorectal cancer [16]. In the present study, a highly selective inhibitor of JAK2, CEP-33779 was used to study JAK2 kinase activity in mouse oocyte maturation.

## Materials and methods

### Chemicals and animals

All chemicals were obtained from Sigma–Aldrich unless otherwise stated. Hormones were obtained from Ningbo Hormone Company (Ningbo, China). CEP-33779 was from Selleck Chemicals. Female Kunming white mice were obtained from Guangdong Medical and Laboratorial Animal Center (Guangzhou, China).

### Oocyte collection and culture

Animal care and handling were conducted in accordance with policies on the care and use of animals promulgated by the Ethical Committee of the Guangdong Medical and Laboratorial Animal Center.

Female Kunming white mice at 6–8 weeks of age were injected intraperitoneally with 7.5 IU of PMSG. The females were killed 45–48 h post-PMSG administration and fully grown, germinal vesicle (GV)-intact oocytes were retrieved from each ovary by puncturing the follicles with a 26 gauge sterile needle.

The collection medium was M-199 with 25 mM HEPES buffer (Invitrogen). To maintain meiotic arrest at the GV stage, collection medium was supplemented with 50 μM 3-isobutyl-1-methyl-xanthine [17]. The GV oocytes were then transferred into M-199 (Invitrogen) supplemented with 10% FBS, 1 IU/ml FSH, 1 IU/ml hCG, 1 mg/ml estradiol, and 0.036 g/l sodium pyruvate, and were cultured in 5% CO_2_ in air at 37°C.

For CEP-33779 treatment, a stock solution of CEP-33779 was diluted in DMSO to 5 mM and stored at −20°C. At the beginning of each culture, the stock solution was diluted with maturation medium for final concentration of 50 μM. The CEP-33779 concentration was determined from preliminary experiments (result not shown) and only the concentration that had a significant effect on the oocyte maturation was used.

To detect spindles and actin, oocytes were collected at 3, 8, 9, and 12 h, respectively. And this experiment was repeated three times.

### Immunohistochemistry

The oocytes were fixed at 37°C for at least 30 min in a stabilizing buffer containing 2% formaldehyde, 0.5% Triton X-100, 1 mM taxol, 10 units/ml aprotinin, and 50% deuterium oxide. They were then washed three times in a washing buffer PBS containing 3 mM NaN_3_, 0.01% Triton X-100, 0.2% non-fat dried milk, 2% normal goat serum, 0.1 M glycine, and 2% BSA) and then left in the washing buffer overnight at 4°C for blocking and permeabilization [18]. Oocytes were incubated for 1 h with antibody of JAK2 (Aviva Systems Biology) for JAK2 immunostaining. For microtubule staining, oocytes were incubated with mouse monoclonal anti-α-tubulin antibody at a final dilution of 1:200 overnight at 4°C. And then oocytes washed three times in the washing buffer. Oocytes were sequentially incubated with FITC–conjugated secondary antibody (Invitrogen) for 1 h at 37°C to visualize JAK2 or microtubules. To label for microfilaments, the oocytes were subsequently incubated with Rhodamine-Phalloidin (molecular probes) (1:200) for 30 min at 37°C. Finally, the oocytes were washed, stained for DNA with Hoechst 33342 (molecular probes), mounted in PBS containing 50% glycerol, as an antifading reagent, and 25 mg/ml NaN_3_ [19] and examined with a fluorescent microscope (Olympus, IX71).

### Statistical analysis

All percentages from at least three repeated experiments are expressed as means ± S.E.M. Data were analyzed by independent sample *t* test. *P*<0.05 was considered statistically significant.

## Results

### Localization of JAk2 in mouse oocyte during meiotic maturation

**Figure 1:**
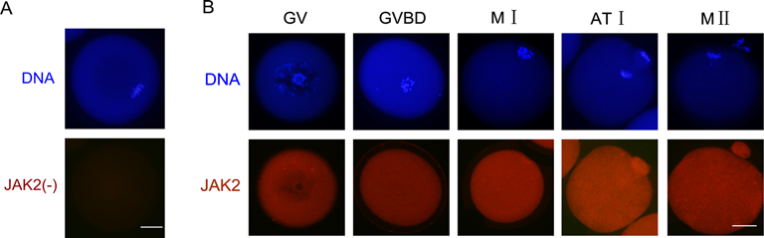
Immunolocalization of JAK2 in mouse oocyte of different meiotic stages. (**A**) The negative control of JAK2 immunolocalization. (**B**) Oocytes were double-stained for JAK2 (red) and DNA (blue) at stage GV (0 h), GVBD (3 h), metaphase I (M I) (8 h), anaphase I (AT I) (9 h), and M II (12 h). The scale bar is 20 μm.

We first examined the localization of JAK2 at different meiotic maturation stages. In the GV stage, most JAk2 was observed in the cytoplasm but less in the nucleus. From GVBD to M II stages, JAK2 still localized in the cytoplasm (Figure 1).

### CEP-33779 treatment results in failure of mouse oocyte maturation

**Figure 2:**
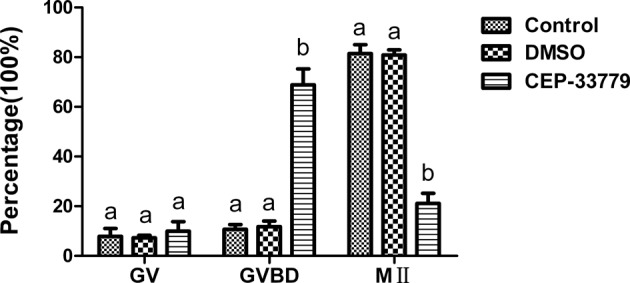
Development of GV oocytes cultured with CEP-33779. Oocytes were cultured without DMSO and CEP-33779 (control, *n*=357), with DMSO control (DMSO, *n*=363), with CEP-33779 (CEP-33779, *n*=382). After 12 h of culturing, the percentage of different meiotic stages were analyzed. Different superscripts depict significant differences between groups in each meiotic stage (*P*<0.05).

Intact GV-oocytes were cultured with CEP-33779 for 12 h to inhibit JAK2 activity during maturation and the first polar body extrusion of oocytes was checked. As shown in Figure 2, a large proportion of the CEP-33779 treated oocytes failed to extrude polar body I, only 21.14 ± 4.03% (*n*=382)of these oocytes reached the M II stage after 12 h *in vitro* maturation, a rate significantly lower than that of non-treated control oocytes (81.44 ± 3.64%, *n*=357). Because of the CEP-33779 dissolved with DMSO, we checked the effects of DMSO on the maturation of oocytes* in vitro*. The rates of maturation *in vitro* were not different between the control and the DMSO groups (80.88 ± 2.08%, *n*=363).

These results indicate that CEP-33779 has a direct effect on the mouse oocyte maturation process and inhibition of JAK2 caused the failure of meiotic maturation.

### CEP-33779 treatment has no effect on spindle structure during mouse oocyte maturation

**Figure 3:**
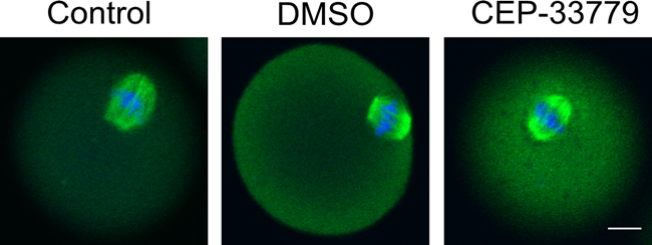
CEP-33779 does not disorder microtubules reorganization in mouse oocytes. Metaphase I(M I) spindle morphology of mouse oocytes were observed during meiosis in control (*n*=77), DMSO (*n*=83), and CEP-33779 (*n*=72) groups. DNA (blue) and tubulin (green) were stained with DAPI and antitubulin antibody, respectively. The scale bar is 20 μm.

As mentioned above, 68.86% oocytes that underwent GVBD could not progress to M II stage. Because the meiotic spindle is the main cellular apparatus responsible for cell division (polar body extrusion), we examined the structure of meiotic spindle. As shown in Figure 3, treatment with CEP-33779 for 12 h did not cause any noticeable difference in spindle configuration relative to untreated controls.

### CEP-33779 treatment disorder microfilaments aggregation and function in mouse oocytes maturation

As the results showed that the 68.86% oocytes occurred GVBD, but could not extrude the polar body treated with CEP-33779. It indicated that CEP-33779 did not affect the GVBD of mouse oocytes. As we know, microfilaments are critical for the migration of meiotic spindle toward the periphery of oocytes [20,21]. Microfilaments are reorganized when meiotic spindle approaching and formed into a special structure named actin cap, which marks the site of polar body extrusion [22].In order to detect whether CEP-33779 inhibits polar body extrusion by destroying the function of microfilaments. We detected the microfilaments in the present study.

**Figure 4:**
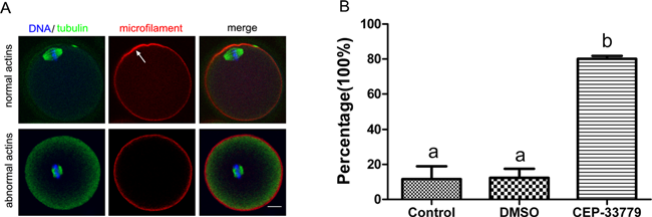
CEP-33779 disrupts microfilaments aggregation in mouse oocytes. (**A**) Location of microfilament (red) in mouse oocytes. Normal microfilaments form the actins cap (white arrow) near the spindle composed of chromosomes (blue) and microtubules (green). Abnormal microfilaments do not form the actins cap and spindle stay at the center of the oocyte. The scale bar is 20 μm. (**B**) The abnormal microfilament percentage in control (*n*=82), DMSO (*n*=88), and CEP-33779 (*n*=77) groups, respectively. Different superscripts depict significant differences between groups (*P*<0.05).

As shown in Figure 4A, the chromatins with microtubules stay in the center of the most oocyte treated with CEP-33779, indicating the microfilaments network responsible for spindle or chromatins movement was dysfunctional. At the same time, microfilaments did not rearrange to form actin caps, showing the organization of microfilaments was disrupted by CEP-33779. Oocytes underwent GVBD in three groups, 80.12% oocytes did not form actin caps and the spindle stayed at the center of oocytes in CEP-33779 group (*P*<0.05), only 11.67 and 12.33% oocytes did so in control and DMSO group (Figure 4B). The results indicated that the JAK2 plays a regulatory role in regulation microfilament function probably.

## Discussion

### The location of JAK2 in mouse oocyte during mouse oocyte maturation

In most of the somatic cells, JAK2 mainly exists in the cytoplasm. In Nb2 cells and rat liver hepatocytes, the JAK2 exists in the cytoplasm and nucleus. However, it only exists in the nucleus of Langerhans cells [23,24]. Our results showed that JAK2 was mainly located in the cytoplasm of oocytes in GV, GVBD, and M II phases. In contrast with the other result, oocytes in the M II phase of JAK2 were only distributed in the nucleus [12]. The different distribution of JAK2 in M II phases of oocyte between the two study results may be because of the different JAK2 antibodies used. However, that study did not detect the distribution of JAK2 in GV and any other the first meiotic phase of oocytes. In oocytes, JAK2 may be distributed not only in nucleus but also in cytoplasm.

### The inhibitor of JAK2 CEP-33779 inhibits the mouse oocyte maturation

In order to study the role of JAK2 in mouse oocyte maturation, JAK2 specific inhibitor CEP-33779 was used to treat oocytes. The results showed that CEP-33779 could significantly decrease the maturation rate of oocytes. The results suggest that JAK2 plays an important role in oocyte maturation. So far, we have not found the literature on the role of JAK2 in oocyte maturation.

### JAK2 do not effect that the spindle structure during the mouse oocyte maturation

The spindle is an important structure of oocytes during meiosis, so we attempt to detect spindle structure to study the effect of JAK2 during the process of oocyte maturation. The microtubules are essential component of meiotic spindle, which manages polar body extrusion and distributes genetic materials equally into the polar body and the remaining oocyte. As the oocytes begin to mature, microtubules polymerize around the chromosomes and eventually assemble into the meiotic spindle. Our results showed that CEP-33779 did not destroy the aggregation of microtubules, there was no significant change in the spindle structure of oocytes. The results showed that JAK2 did not affect the aggregation of microtubules during the mouse oocyte maturation.

### CEP-33779 disorders microfilaments in mouse oocytes

Microfilaments are required for chromosomes or spindle migration to the cortex of oocytes and are essential for oocyte asymmetric cell divisions. As one of the important cytoskeleton in somatic cells, microfilaments play an important role in cell morphology, migration, signal transduction, and so on. It has been reported that blockage of JAK2/STAT3 pathway up-regulated the expression of p27 [25]. And the up-regulation of p27 led to the reduction in cell migration because of the damage of microfilaments [26]. But some data from the literature suggest that the inhibition of the JAK2/STAT3 pathway is not upstream in the cascades that lead to cucurbitacin-dependent actin aggregation [27]. In the present study, microfilaments could not aggregate to form actin cap with the CEP-33779. The results showed that JAK2 affected the microfilaments function during mouse oocytes maturation. And the results are consistent with JAK2 localization in cytoplasm. However, the mechanism by which JAK2 affects microfilament function remains to be further studied.

In the present study, using a special inhibitor of JAK2, we found that CEP-33779 significantly affected the mouse oocyte maturation. We also showed that JAK2 inhibition caused aberrant actin aggregation and the failure of spindle positioning, which might have contributed to a failure of mouse oocyte polar body extrusion. Therefore, JAK2 may regulate actin-mediated spindle positioning to participate in the mouse oocyte maturation.

## Abbreviations

AOM/DSS: azoxymethane/dextran sodium sulfate
CEP-33779: a selective inhibitor of JAK2
FSH: follicule stimulating hormone
GVBD: germinal vesicle breakdown
GV: germinal vesicle
hCG: human chorionic gonadotropin
H3Y41: throsine 41 on histone H3
IU: international unit
JAK2: janus kinase 2
M-199: medium 199
M II: metaphase II
Nb2: T-lymphoma cell line
PMSG: pregnant mare serum gonadotrop
STAT: signal transducer and activator of transcription
